# Coronary Ostial Angioplasty for Takayasu Arteritis Using External Iliac Artery Graft: 1-year Follow-up

**DOI:** 10.1016/j.atssr.2024.12.005

**Published:** 2024-12-25

**Authors:** Takayuki Kawamura, Shuichiro Takanashi, Ken Chen, Yosuke Mukae, Kenta Zaikokuji, Tomoki Shimokawa, Mitsuaki Isobe, Tomohiro Iwakura

**Affiliations:** 1 Department of Cardiovascular Surgery, Adult, Sakakibara Heart Institute, Tokyo, Japan; 2 Department of Cardiac Surgery, Kawasaki Saiwai Hospital, Kanagawa, Japan; 3 Department of Cardiovascular Surgery, Teikyo University School of Medicine, Tokyo, Japan; 4 Sakakibara Heart Institute, Tokyo, Japan

## Abstract

**Purpose:**

The optimal treatment of coronary stenosis caused by Takayasu arteritis (TAK) remains controversial.

**Description:**

We present 3 cases in which an innovative approach with an external iliac artery (EIA) patch was employed for ostial angioplasty in young patients with TAK. We determined the factor influencing the success of this technique, which was similar to those having an impact on other treatments.

**Evaluation:**

All patients underwent the procedure successfully, demonstrating favorable outcomes, such as improved blood flow and symptom relief, compared with those observed after traditional revascularization methods. We describe the technique for EIA patch angioplasty.

**Conclusions:**

Considering the anatomic and pathologic factors, coronary ostial angioplasty with an EIA patch may offer a more effective and durable solution for managing coronary ostial stenosis in patients with TAK. However, despite the potential of this technique, further studies and long-term follow-up are essential to validate its efficacy and safety.

## Visual Abstract

**Figure:**
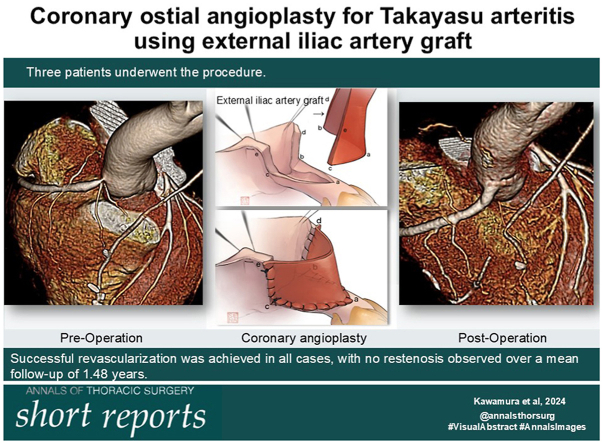

## Technology

Takayasu arteritis (TAK) is a nonspecific inflammatory disease that primarily affects the aorta and its major branches, notably the coronary arteries, commonly in young women.^1^ The coronary arterial involvement rate is 9.0% in Japanese patients with TAK.^2^ High-dose glucocorticoids manage active inflammation, and tocilizumab may reduce recurrence.^3^

The optimal revascularization approach for coronary artery stenosis remains unclear. Percutaneous coronary intervention (PCI) has a high incidence of short-term restenosis, and coronary artery bypass grafting (CABG) may have complications, such as subclavian artery stenosis and long-term restenosis.

The concept of reconstructing the left main trunk and aortic wall with external iliac artery (EIA) grafts is to rebuild the roof of the main trunk of the left coronary artery higher with EIAs and to replace the inflammation-altered aortic wall adjacent to the coronary artery orifice with extensive EIAs. We previously reported a method for coronary ostial angioplasty with an EIA patch for a TAK patient.^4^ We have subsequently used this method in 2 more patients with longer follow-up. Here, we describe the continued exploration of this approach, which aims to evaluate its safety and efficacy for advancing coronary ostial treatment of TAK.

## Technique

Patient 1 involved a 21-year-old woman diagnosed with TAK who presented with chest pain. Coronary angiography and computed tomography (CT) confirmed occlusion of the left main coronary artery (LMCA) and stenosis of the ostium of the right coronary artery (RCA). Corticosteroid therapy began with methylprednisolone pulse, followed by prednisolone tapered during 2 months. Subsequently, the patient underwent minimally invasive CABG with a left internal thoracic artery anastomosis to the left anterior descending artery. However, a stress electrocardiogram performed before the second operation showed extensive ST depression. Postoperative coronary angiography revealed stenosis at the proximal and distal sites of graft anastomosis. It is possible that not only technical failure but also active arteritis contributed to the progression of lesions at the surgical site.

Methylprednisolone pulse therapy was repeated, and tocilizumab treatment was added to the corticosteroid. ^13^N-ammonia positron emission tomography revealed extensive myocardial ischemia in the left ventricle.

Intervention at the coronary artery distal anastomosis was not performed because of its effect on inflammatory vessels and narrow perfusion zone. To address these concerns, bilateral coronary artery ostial angioplasty was performed following the technique reported by Arai and colleagues.^5^ We made a 40-mm oblique incision for retroperitoneal harvesting of the left EIA with a diameter of 8 mm and a length of 60 mm, which was employed as patch material instead of the superficial femoral artery. Subsequently, this section was replaced with a 60-mm vascular prosthesis (Triplex Advanced, 8 mm; Terumo).

**Figure 1:**
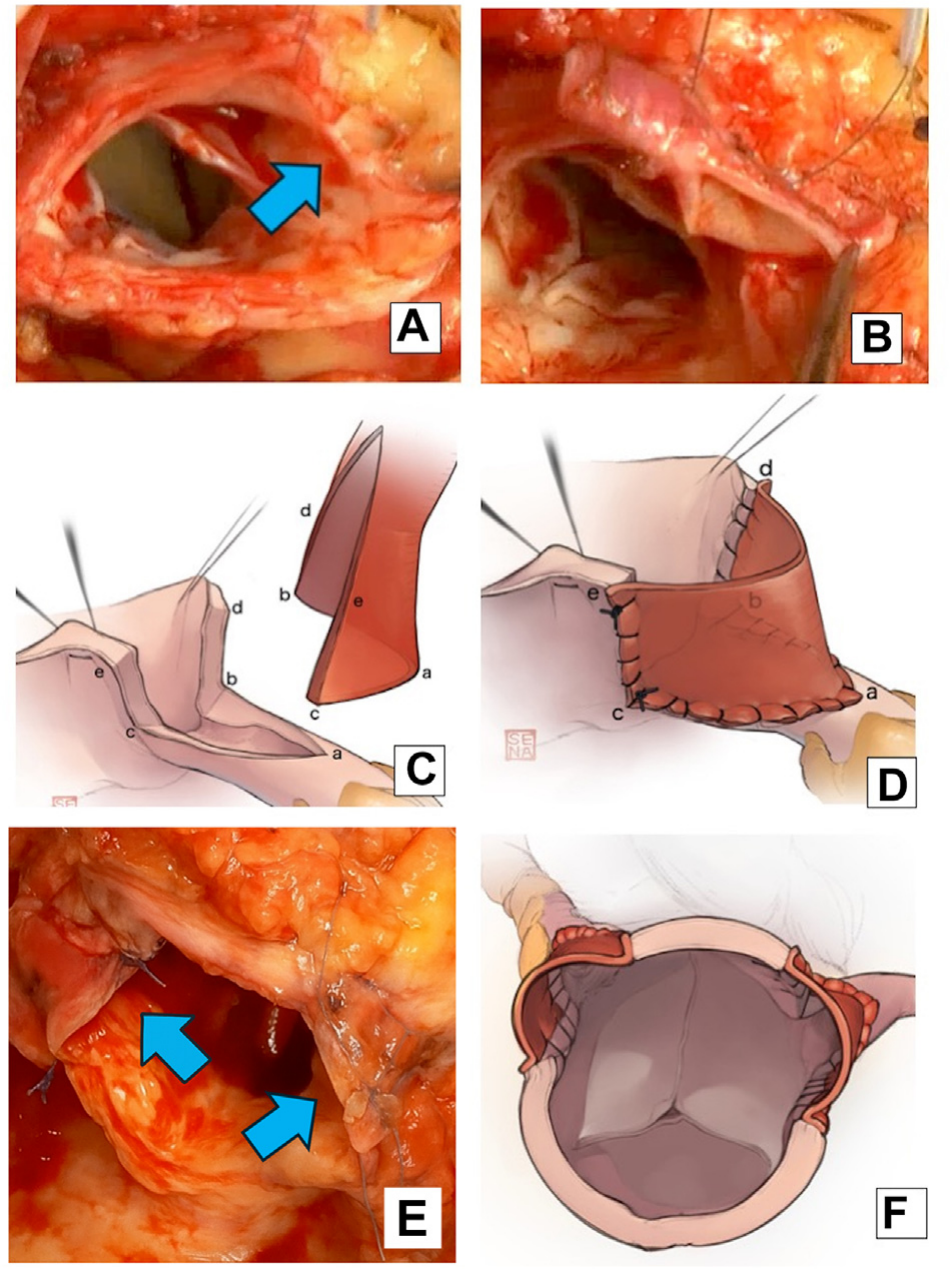
(A) Coronary ostium stenosis with a thickened aortic wall. An incision was made perpendicular to the right coronary artery in a lesion-free area (blue arrow). (B-D) An external iliac artery patch was cut longitudinally and anastomosed from the right coronary artery to the aortic wall with a 7-0 monofilament suture. (E, F) The external iliac artery patch was sutured to enlarge the coronary artery ostium (blue arrows).

Cardiopulmonary bypass was established at the ascending aorta and right atrium, with a vent cannula applied to the right upper pulmonary vein. We used retrograde myocardial protection from the coronary sinus and selective myocardial protection. The left internal thoracic artery and aorta were clamped. The details of this technique have been described previously.^4^ An incision was made in the aortic wall perpendicular to the c oronary ostia, positioned 15 mm above both the right and left ostia. A 1-cm incision was made on the superior side of the RCA ostium, starting at the coronary ostium, for anastomosis with the RCA (Figures 1A, 1B).

The EIA graft was incised longitudinally to create a rectangular patch. The cut coronary artery was stitched to roughly match the circumference of the EIA patch. The incision lines of the aortic wall and long axis of the EIA patch were aligned.

The patch was anastomosed to the RCA and extended to the aortic wall. A 7-0 monofilament suture secured the patch at the distal end of the RCA incision (Figures 1C, 1D). The incised coronary artery was sutured to match the circumference of the EIA patch. We aligned the aortic wall incision line and EIA patch along the aortic long axis using a 6-0 monofilament suture. We performed LMCA coronary angioplasty using the same approach. Both coronary ostia were enlarged by 1 cm with an EIA graft (Figures 1E, 1F). The aortotomy was anastomosed with a 5-0 monofilament suture.

**Figure 2:**
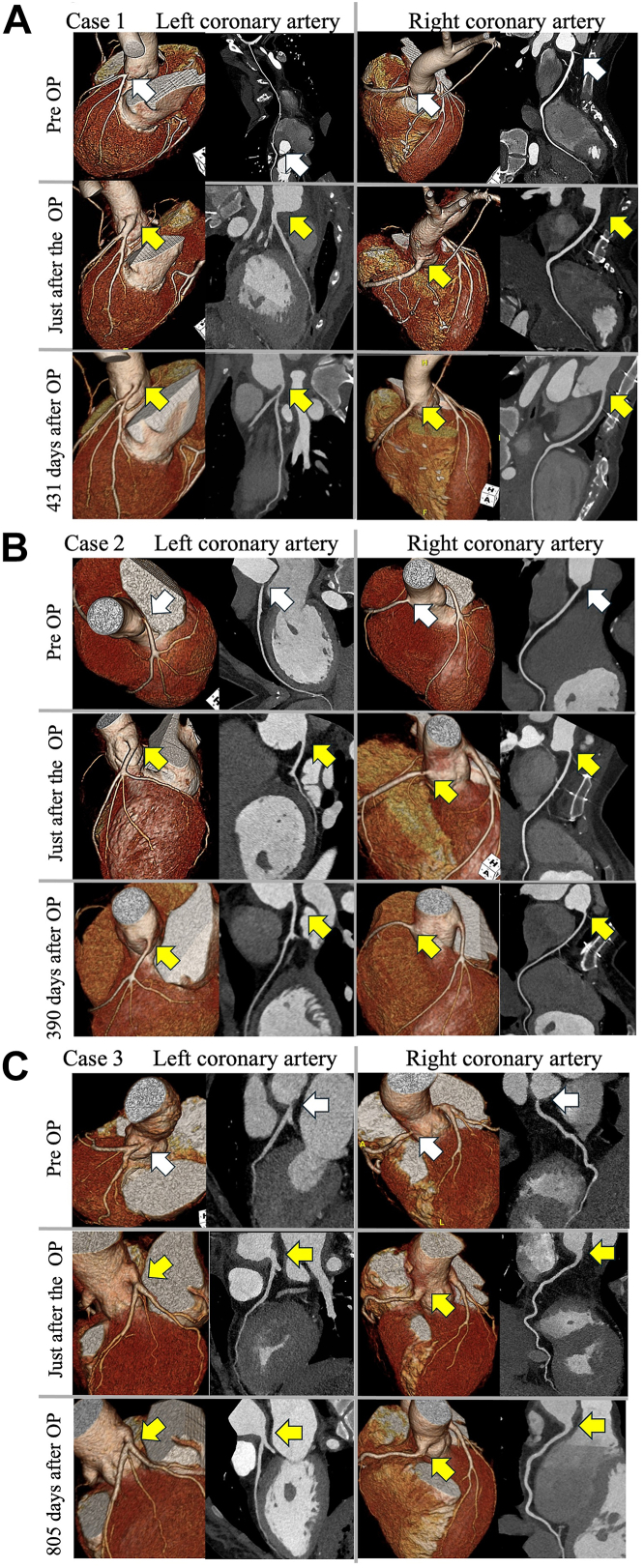
Preoperative and postoperative multiplanar reconstruction computed tomography images of each coronary artery. (A) Patient 1. (B) Patient 2. (C) Patient 3. White arrows indicate coronary ostial lesions. Yellow arrows indicate enlargement of the left main coronary artery ostium by an external iliac artery patch. (OP, operation; Pre OP, preoperative.)

Histologic examination of the EIA patch showed no abnormalities or inflammation. The patient resumed prednisolone (15 mg) on postoperative day 3. Postoperative CT showed no stenosis at the coronary ostium or excised artery site. The patient was discharged on postoperative day 14, and methotrexate and tocilizumab administration resumed. After 431 days, a follow-up CT scan showed no restenosis (Figure 2A), and the chest pain did not return.

Patient 2 involved a 24-year-old woman with TAK admitted to another hospital for acute myocardial infarction. Coronary angiography revealed 99% LMCA ostium stenosis and 75% RCA stenosis. Venoarterial extracorporeal membrane oxygenation and Impella (Abiomed) were initiated. Balloon angioplasty was performed as an emergency for the LMCA, and prednisolone (50 mg/d) was administered. The patient’s cardiac function improved, allowing venoarterial extracorporeal membrane oxygenation and Impella withdrawal. One month later, exercise myocardial scintigraphy indicated extensive LMCA ischemia, and coronary CT showed progressive stenosis (75%-90%) in the LMCA. The patient was administered prednisolone, which was tapered to 10 mg/d, and tocilizumab (162 mg/wk) twice.

**Figure 3:**
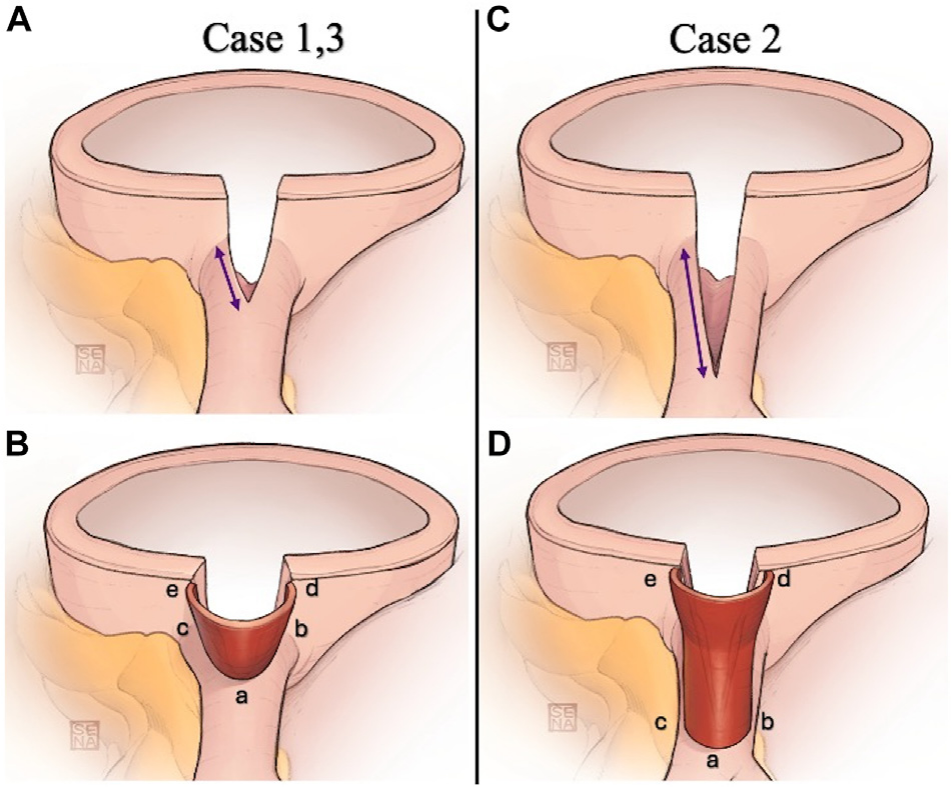
(A, B) If the coronary incision was shorter than the half-circumference of the external iliac artery (EIA), the EIA patch was aligned vertically with the coronary incision (patient 1, 3). (C, D) If it was longer, the EIA patch was joined horizontally (patient 2).

The patient underwent LMCA coronary angioplasty. The lesion required a longer 1.5-cm incision toward the LMCA. As this exceeded half the EIA circumference, we rotated the EIA patch 90° to prevent kinking and to enlarge the coronary ostium (Figures 3C, 3D). Immediately and after 390 days, follow-up CT showed no restenosis (Figure 2B), and the patient was not experiencing any chest pain.

Patient 3 involved a 15-year-old girl with TAK who complained of chest pain at rest and was referred to our hospital. The patient had received prednisolone (10 mg/d), methotrexate (14 mg/wk), and tocilizumab (162 mg/wk) for 6 months. The patient had a history of Sjögren syndrome. Coronary CT showed 75% RCA and 90% LMCA ostial stenosis, bilateral subclavian and carotid artery occlusion, and narrowing of the thoracic descending aorta.

The patient underwent bilateral coronary ostial angioplasty by the same method as in cases 1 and 2.^4^ Contrast-enhanced CT findings immediately, 1-year after operation, and after 805 days showed no complications at the anastomosis site (Figure 2C). The patient did not experience any chest pain.

## Clinical Experience

This procedure may be considered for young patients with coronary ostial stenosis caused by TAK, particularly when the lesions do not respond to immunosuppressive therapy. However, its applicability may be constrained by distal coronary stenosis or active disease in the EIA graft. In cases of TAK with active inflammation, this approach serves as a viable alternative to bypass grafting.

## Comment

We report 3 cases using our pioneering EIA patch approach for coronary artery ostial angioplasty in TAK patients (Table). TAK induces inflammation and structural changes in the ascending aorta, with granulomatous inflammation affecting the vascular adventitia and the outer part of the media.^1^ Coronary artery stenosis is common in TAK patients,^2^^,^^6^ often being manifested as ostial lesions. Whereas various revascularization methods exist, PCI has a high restenosis rate.^7^^,^^8^ CABG raises long-term patency concerns. Thus, rather than PCI or CABG, coronary ostial angioplasty may be a promising option for treating coronary disease for TAK patients. For patch plasty, the saphenous vein patch lacks sufficient width for use as an ostial patch and carries a higher risk of dilation, loss of elasticity, and intimal hyperplasia. Long-term patency may not be expected compared with the EIA patch. Although autologous pericardium^9^ and bovine pericardium are also used, these materials are less flexible and thinner than the EIA. They may not provide the same long-term structural rigidity as the EIA.

**Table:** Clinical Profile of Patients

Clinical Profile	Patient 1	Patient 2	Patient 3
Age, y	21	24	15
Sex	Female	Female	Female
Takayasu arteritis onset	Chest pain	Shortness of breath	Syncope
Coronary artery lesion	LMCA RCA ostium	LMCA	LMCA RCA ostium
Another vascular lesion	Ascending aorta to aortic arch	Ascending aorta	Ascending aorta Bilateral subclavian artery Bilateral carotid artery Descending abdominal aorta
Immunosuppressive treatment			
Initial treatment	Steroid pulse	Steroid pulse	Steroid pulse
before operation	Tocilizumab Prednisolone (15 mg) Methotrexate	Tocilizumab Prednisolone (10 mg)	Tocilizumab Prednisolone (15 mg) Methotrexate
Perioperative period	Prednisolone	Prednisolone	Prednisolone
After discharge	Tocilizumab Prednisolone (15 mg) Methotrexate	Tocilizumab Prednisolone (10 mg)	Tocilizumab Prednisolone (15 mg) Methotrexate

LMCA, left main coronary artery; RCA, right coronary artery.

Although the superficial or common femoral artery is an option for the ostial patch, it may cause stenosis after harvesting and compromise future access. The EIA possesses adequate thickness and is easier to handle and more resistant to bending than other materials. The EIA can be cut to the required size and length, even for bilateral coronary angioplasty. Replacing the coronary artery almost entirely significantly reduces the risk of restenosis at the coronary ostium.

Using the EIA patch in an upright position not only reduces the flexion of the folded-back portion of the coronary artery ostium but also capitalizes on the 3-dimensional nature of the patch (Figures 3A, 3B). This 3-dimensional design enables reconstruction of the coronary artery ostium with a natural bulge, facilitating optimal blood flow dynamics. As in patient 2, there was concern that the longer coronary artery incision might lead to insufficient graft material if the EIA patch were to be aligned vertically. To address this, we rotated the EIA patch 90°, to a horizontal position, matching the long axis of the incision (Figures 3C, 3D).

Immunosuppressive treatment before surgical treatment is also important. Recurrence of inflammation during the tapering of corticosteroids is reported to be ≥70% in patients with TAK.^10^ Surgical treatment should be performed after the active inflammation is calmed down to prevent poor vascular outcomes. Furthermore, high corticosteroid doses increase perioperative complication risk. We employed short-term tocilizumab application before operation in all cases. The remission induction rate has been reported to exceed 70% after tocilizumab treatment in TAK.^3^ However, one of the problems with perioperative tocilizumab use is that the agent almost completely suppresses inflammatory biomarkers, including C-reactive protein. To avoid this, we stopped tocilizumab injections 3 to 4 weeks before operation in these cases, considering the prolonged effect of the biologic agent.

In general, it is extremely rare that TAK involves iliac arteries. Preoperative imaging confirmed the absence of inflammatory involvement at the EIA area. Pathologic examination of harvested grafts also confirmed the absence of inflammatory cell infiltration.

A considerable portion of the aortic wall associated with the coronary artery is replaced with an EIA, which is less vulnerable to aortitis recurrence. We also intensified immunosuppressive treatment before operation. However, inducing inflammation at the anastomosis site remains possible because of the pathophysiologic process of TAK, regardless of the surgical procedure used.

In conclusion, considering both anatomic and pathologic factors, coronary ostial angioplasty with EIA may appear more rational than CABG or PCI in patients with TAK. Our midterm outcomes, with a mean follow-up of 1.48 years, were favorable. Long-term follow-up to validate the efficacy of this procedure is warranted.

## Freedom of Investigation

The technology tested in this study was donated for the purposes of this research. The authors had full control over the design of the study, the methods used, the outcome parameters, the analysis of the data, and the production of the written report.

## Disclaimer

The Society of Thoracic Surgeons, The Southern Thoracic Surgical Association, and *The Annals of Thoracic Surgery Short Reports* neither endorse nor discourage the use of the new technology described in this article.
